# Reactive Oxygen Species Alleviate Cell Death Induced by Thaxtomin A in *Arabidopsis thaliana* Cell Cultures

**DOI:** 10.3390/plants8090332

**Published:** 2019-09-06

**Authors:** Fatima Awwad, Guillaume Bertrand, Michel Grandbois, Nathalie Beaudoin

**Affiliations:** 1 Centre SÈVE, Département de Biologie, Université de Sherbrooke, Sherbrooke, QC J1K 2R1, Canada; 2 Groupe de Recherche en Biologie Végétale, Département de Chimie, Biochimie et Physique, Université du Québec à Trois-Rivières, Trois-Rivières, QC G9A 5H7, Canada; 3 Institut de Pharmacologie de Sherbrooke, Département de Pharmacologie et Physiologie, Université de Sherbrooke, Sherbrooke, QC J1H 5N4, Canada (G.B.) (M.G.)

**Keywords:** cell wall rigidity, H_2_O_2_, programmed cell death, thaxtomin A

## Abstract

Thaxtomin A (TA) is a cellulose biosynthesis inhibitor synthesized by the soil actinobacterium *Streptomyces scabies*, which is the main causal agent of potato common scab. TA is essential for the induction of scab lesions on potato tubers. When added to *Arabidopsis thaliana* cell cultures, TA induces an atypical programmed cell death (PCD). Although production of reactive oxygen species (ROS) often correlates with the induction of PCD, we observed a decrease in ROS levels following TA treatment. We show that this decrease in ROS accumulation in TA-treated cells is not due to the activation of antioxidant enzymes. Moreover, *Arabidopsis* cell cultures treated with hydrogen peroxide (H_2_O_2_) prior to TA treatment had significantly fewer dead cells than cultures treated with TA alone. This suggests that H_2_O_2_ induces biochemical or molecular changes in cell cultures that alleviate the activation of PCD by TA. Investigation of the cell wall mechanics using atomic force microscopy showed that H_2_O_2_ treatment can prevent the decrease in cell wall rigidity observed after TA exposure. While we cannot exclude the possibility that H_2_O_2_ may promote cell survival by altering the cellular redox environment or signaling pathways, our results suggest that H_2_O_2_ may inhibit cell death, at least partially, by reinforcing the cell wall to prevent or compensate for damages induced by TA.

## 1. Introduction

Reactive oxygen species (ROS) are involved in several plant physiological processes, including growth and development, stress and defense responses, tissue differentiation, and senescence. ROS can regulate gene expression and participate as secondary messengers in various signaling pathways [1,2,3]. Plant cells can produce ROS enzymatically to fulfill specialized function or in response to a wide variety of stimuli. ROS production can also occur non-enzymatically, during photosynthesis and respiration, through electron transport chain reactions occurring in chloroplasts and mitochondria [1,3,4]. While basal ROS levels are essential to maintain normal cell functions, stress conditions can greatly enhance their accumulation up to toxic levels and cause important oxidative damage to cells. To maintain an adequate redox balance and prevent ROS-mediated damage, plant cells have evolved different anti-oxidative strategies involving various non-enzymatic and enzymatic ROS scavengers [2,3,5]. 

Hydrogen peroxide (H_2_O_2_) is a type of ROS involved in central physiological processes and signaling pathways within and between plant cells [6,7]. For instance, H_2_O_2_ has been implicated in the regulation of stomatal movements [8], seed germination [9], root system development, root hair growth [10], pollen tube formation [11], and reinforcement of the cell wall [12,13,14]. Generation of H_2_O_2_ can be mediated by enzymes such as cell wall peroxidases [15], cell membrane NADPH-dependent oxidases [16], and amine oxidase [17]. Plant cells exposed to stress can produce a burst of ROS mainly composed of H_2_O_2_ [18]. Such H_2_O_2_ production has been detected in response to various abiotic and biotic stresses, such as heat stress [19], wounding [20], salt stress [21], and response to a bacterial or a fungal pathogen [22,23,24]. Plant pathogen recognition also involves a rapid ROS accumulation (the so-called “oxidative burst”) associated with the activation of defense-related programmed cell death (PCD) known as the hypersensitive (HR) cell death [25]. Production of H_2_O_2_ is commonly associated with most types of developmental or stress-induced PCD [6]. Moreover, H_2_O_2_ itself can activate PCD [26]. At high concentrations, H_2_O_2_ is highly toxic to all living cells. H_2_O_2_ can react with various cellular components, causing extensive damages that may lead to cell death. Hence, H_2_O_2_ production has been involved in direct anti-pathogen roles [18]. However, high levels of H_2_O_2_ also induce the synthesis of antioxidant molecules that can protect cells from oxidative damage. Consequently, it is now widely accepted that H_2_O_2_ produced during PCD mainly plays a role as an important signaling component of complex PCD regulatory pathways rather than being the direct cause of cell death [2]. 

Using *Arabidopsis thaliana* cell suspensions, it was shown that the phytotoxin thaxtomin A (TA) can induce a form of PCD that is not associated with ROS production and the activation of mitogen activated protein kinase (MAPK) signaling cascades [27,28]. TA is synthesized by the phytopathogen *Streptomyces scabies* (syn. *scabiei*), which is a soil actinobacterium causing potato common scab [29,30,31]. This phytotoxin is essential for the formation of disease symptoms characterized by scab-like suberized cell layers on the surface of potato tubers [30,32,33,34]. TA inhibits cellulose biosynthesis, which may facilitate pathogen entry through the cell walls of potato cells [31,35,36]. In *A. thaliana* seedlings, TA treatment also inhibits root growth and causes root swelling [36,37]. Recently, it was shown that TA activates Enhanced Disease Susceptibility 1 (EDS1)-dependent and Phytoalexin Deficient 4 (PAD4)-dependent defense responses independently of SA production [38]. EDS1 and PAD4 are important regulators of plant innate immunity and were shown to be essential for disease resistance. During plant pathogen interactions, EDS1/PAD4 form complexes that activate defense gene expression, which leads to plant immunity and/or localized cell death [39,40,41,42]. Activation of EDS1/PAD4-mediated pathway by TA does not depend on ROS production [38].

Considering the documented implication of ROS in most plant PCD pathways, the fact that ROS production is not stimulated by TA is quite intriguing. In this work, we studied this question using *A. thaliana* cell suspensions. We first showed that ROS production was diminished by TA treatment. We investigated the possible role of antioxidant enzymes catalase (CAT), ascorbate peroxidase (AXP), and superoxide dismutase (SOD) in controlling ROS accumulation in response to TA. We found that addition of H_2_O_2_ to cell cultures prior to TA treatment protected cells from TA-induced cell death. Measurements of cell surface mechanics using atomic force microscopy (AFM)-based force microscopy showed that, while TA treatment decreased cell wall stiffness, the addition of H_2_O_2_ alone or in combination with TA increased cell wall rigidity. This suggests that ROS may, at least partially, inhibit TA-induced PCD by preventing or compensating for cell wall damages induced by TA.

## 2. Results

### 2.1. Reduced Accumulation of ROS Was Not Asssociated with Increased Antioxidant Enzyme Activity

**Figure 1:**
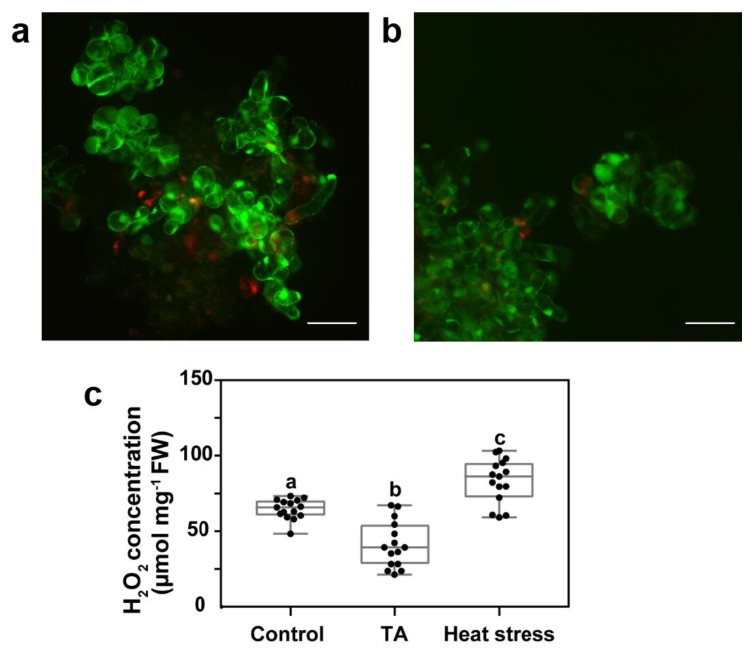
Detection of a reactive oxygen species (ROS). (**a**) ROS were detected using H_2_DCFDA (green) in *Arabidopsis* suspension cells treated for 24 h with 0.1% methanol (Control) or (**b**) with 1 µM thaxtomin A (TA). Scale = 100 µm. (**c**) H_2_O_2_ concentration was measured in µmol·mg^−1^ fresh weight (FW) using a modified FOX assay in cells treated for 24 h with 0.1% methanol (Control), 10 µM thaxtomin A (TA), or at 45 °C for 30 min (Heat stress). Values represent 15 measurements from three independent experiments. The three groups were compared using Dunnett’s multiple comparison test. Different letters indicate statistically significant differences, *p* < 0.05.

Previous work has shown that TA-induced PCD in *Arabidopsis* cell suspension cultures was not associated with the production of ROS [27,28]. We first evaluated the level of ROS production before and after TA-treatment in the *Arabidopsis* suspension cultures used in the present study. Since TA was diluted in methanol, control cells were treated with the same volume of methanol, with a final concentration of 0.1% methanol or less. As reported before, this methanol concentration had no impact on cell viability (Suppl. Figure S1, [27,28]) or ROS production [28,43]. TA-treated and control cells were incubated with H_2_DCFDA, which is a general oxidative stress indicator that emits fluorescence in the presence of ROS. We observed some H_2_DCFDA fluorescence in control cells, which indicates basal ROS production (Figure 1a). However, there was less fluorescence detected in TA-treated cells, which indicates that TA did not stimulate ROS production and appeared to decrease ROS abundance (Figure 1b). We then quantified H_2_O_2_ production in cell cultures using ferrous oxidation-xylenol orange (FOX) modified assay (Figure 1c) [44,45]. There was a significantly lower production of H_2_O_2_ in TA-treated cells when compared to the control cells. Heat stress of cell cultures was used as a positive control of H_2_O_2_ production [19]. These results showed that TA-induced cell death was not associated with the production of ROS. In fact, H_2_O_2_ accumulation in TA-treated cell cultures was significantly decreased below control levels.

**Figure 2:**
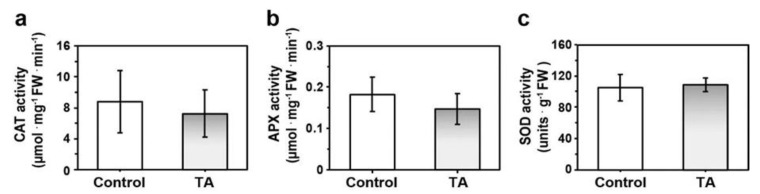
Antioxidant enzyme activity. *Arabidopsis* cell suspensions were treated with 0.1% methanol (Control) or 1 µM thaxtomin A (TA) for 24 h. (**a**) Catalase (CAT) activity in µmol ·mg^−1^ fresh weight (FW)·min^−1^. Each value is the mean of two samples from three biological replicates ± SD (*n* = 6). (**b**) Ascorbate peroxidase (APX) activity in µmol·mg^−1^ FW·min^−1^. (**c**) Superoxide dismutase (SOD) activity in units·g^−1^ FW. (**b**) and (**c**) each value is the mean of three samples per condition ± SD. Data was analyzed using the *t*-test followed by the Holm-Šídák method with alpha = 0.05. No significant difference was detected (*p* < 0.05).

Plants can use diverse mechanisms to control abnormal ROS levels and prevent their deleterious effects on plant cells. One of these mechanisms is the expression or activation of antioxidant enzymes that can scavenge ROS, such as catalase (CAT), ascorbate peroxidase (APX), and superoxide dismutase (SOD) [5]. We quantified these enzyme activities in cell extracts from *Arabidopsis* cell cultures treated or not with TA. The average activity of CAT and APX were lower in TA-treated cells than in control cells, but these differences were not statistically different (Figure 2). Hence, a decrease of ROS accumulation by TA did not involve the activation of these typical antioxidant enzymes.

### 2.2. Hydrogen Peroxide Protected Arabidopsis Cells from TA-Induced Cell Death 

**Figure 3:**
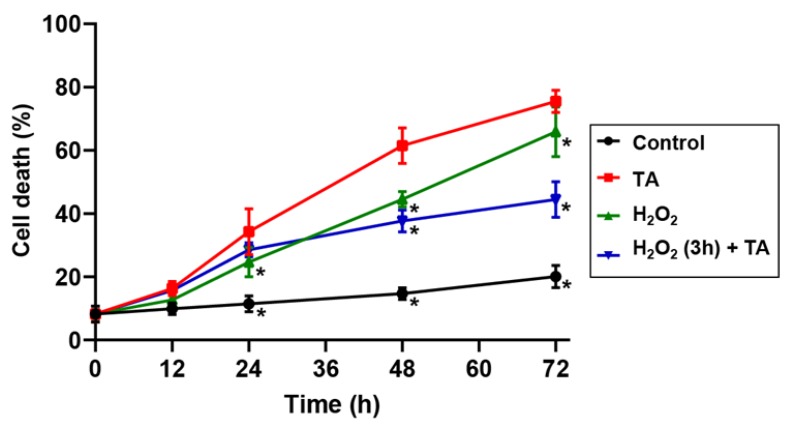
Induction of cell death by thaxtomin A and H_2_O_2_. Percentage of cell death in *Arabidopsis* suspension-cultured cells 48 h after treatment with 0.1% methanol (Control, black circle), 1 µM thaxtomin A (TA, red square), 1 mM H_2_O_2_ (green triangle), and 3 h-pretreatment with 1 mM H_2_O_2_ followed by 1 µM TA (blue inverted triangle). Cells were counted in groups of 500 and the mean ± SD was calculated from three replicates. Data was analyzed using one-way ANOVA. A significant difference with TA treatment for each time point (*p* < 0.05) is indicated by an asterisk (*).

To determine whether reduction of H_2_O_2_ production by TA was associated with the induction of cell death, we added H_2_O_2_ to *Arabidopsis* cell suspension cultures prior to TA treatment. The percentage of dead cells was evaluated in the control cells, after TA treatment, H_2_O_2_ treatment, and a combination of both treatments. We tested two different concentrations of H_2_O_2_ (1 and 10 mM) and a different duration of treatment or pre-treatment to evaluate the effect of H_2_O_2_ on TA-induced cell death (Figure 3, Suppl. Figure S2). The addition of TA alone led to a drastic increase in cell death with close to 61% of dead cells within 48 h when compared to control cell cultures, which had less than 15% of dead cells. Treatment of cell cultures with 1 mM and 10 mM H_2_O_2_ for 48 h induced 44% and 95% of cell death, respectively. For both H_2_O_2_ concentrations, the induction of cell death when H_2_O_2_ and TA were added simultaneously was equivalent to a treatment with H_2_O_2_ alone (data not shown). Pretreatment of cell cultures with 10 mM H_2_O_2_ prior to TA addition led to a significant decrease in cell death when compared to the H_2_O_2_ treatment alone, but the proportion of cell death was comparable to that observed in TA-treated cells (Suppl. Figure S2). When cells were pretreated with 1 mM H_2_O_2_ 3 h prior to TA addition, the proportion of dead cells was significantly decreased down to 38% when compared to 61% in TA-treated cells (Figure 3). These results demonstrate that H_2_O_2_ at this concentration can prevent cell death caused by TA in *Arabidopsis* cell suspension cultures.

### 2.3. Increased Cell Wall Rigidity by H_2_O_2_ was Maintained after TA Treatment

**Figure 4:**
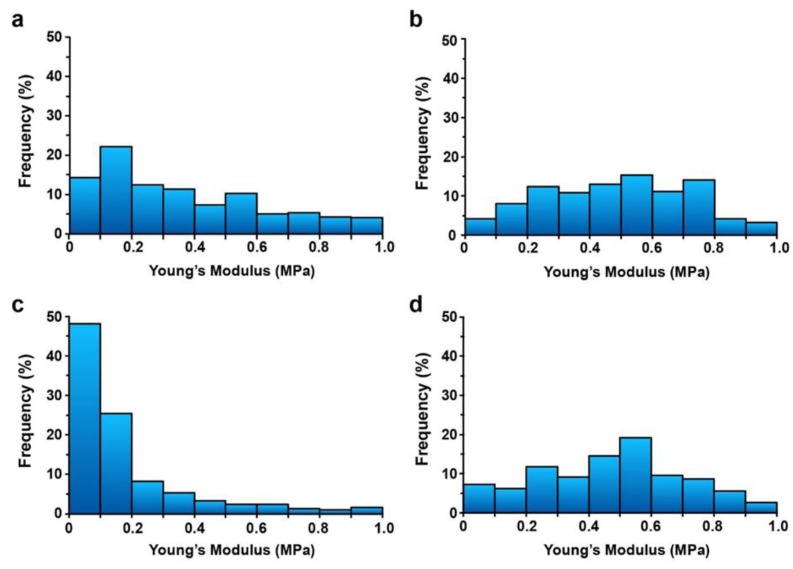
Measurements of cell wall rigidity by Atomic Force Microscopy (AFM). Force curves were recorded at the surface of three to five cells per condition in order to extract Young’s modulus value. Each graph represents the distribution of the relative frequency (%) of Young’s modulus values measured on the cell surface in each condition. *Arabidopsis* cell suspension were treated for 24 h with: (**a**) control (0.1% methanol), (**b**) 1 mM H_2_O_2_, (**c**) 1 µM thaxtomin A (TA), and (**d**) pretreated for 3 h with 1 mM H_2_O_2_ prior to TA treatment.

Treatments of *Arabidopsis* cell cultures with TA or H_2_O_2_ can both alter the cell wall composition and organization. To investigate the mechanical properties of the cell surface of suspension-cultured cells after TA or H_2_O_2_ treatment, we used atomic-force microscopy (AFM) to quantify the elastic modulus (Young’s modulus) of the cell wall, as described before [46]. We compared the overall cell wall stiffness between different cells grown in the same conditions but exposed to different treatments. The distribution of Young’s modulus values in control cells was spread almost evenly in the range of 0.1 to 0.6 MPa, with 14% of values below 0.1 MPa and 49% of values below 0.3 MPa (Figure 4a). In TA-treated cells, 48% of Young’s modulus values were below 0.1 MPa and 82% below 0.3 MPa, which indicates a marked decrease in the stiffness of the cell wall attributed to inhibition of cellulose synthesis (Figure 4c). In contrast, cells treated with H_2_O_2_ alone showed an increase in cell wall stiffness with only 25% of Young’s modulus values distributed below 0.3 MPa and almost half (48%) of the values found above 0.5 MPa (Figure 4b). Similarly, cell walls of cells treated with both H_2_O_2_ and TA showed an increased stiffness with 25% of values below 0.3 MPa and 46% of Young’s modulus values higher than 0.5 MPa (Figure 4d). These results show that cell treatment with 1 mM H_2_O_2_ increased cell wall stiffness in *A. thaliana* cell cultures, and this enhanced rigidity was maintained even after TA treatment.

## 3. Discussion

ROS are key players in signaling and defense responses in plants [47,48,49]. They are generally produced during the induction of PCD activated by both abiotic and biotic stress, where they play a role as second messengers in PCD signaling pathways. The inhibitor of cellulose synthesis thaxtomin A (TA) can activate PCD without increasing ROS production in *Arabidopsis* cell suspensions [27,28]. In this case, we showed that there was less ROS detected by H_2_DCFDA fluorescence in TA-treated suspension-cultured cells (Figure 1a,b) than in control cells. Quantification of H_2_O_2_ levels using a FOX modified assay confirmed a significantly lower production of H_2_O_2_ in TA-treated cells compared to the control cells (Figure 1c). Since production of H_2_O_2_ in the first 4 h after addition of TA to cell cultures was similar to that of untreated cultures [28], the decrease in H_2_O_2_ levels 24 h after TA treatment suggests that TA activated biochemical or molecular mechanisms which could either scavenge ROS or inhibit their production. Our results show that the reduction in ROS production was not due to the activation of the antioxidant enzymes CAT, APX, or SOD (Figure 3). This supports previous results showing that expression of the corresponding CAT, APX, and SOD genes was not induced or repressed by TA treatment in *Arabidopsis* cell suspensions [50]. However, it is possible that TA induced other ROS scavenging mechanisms, such as the activation of other antioxidant enzymes (e.g., gluthatione peroxidase, peroxiredoxins, etc. [5]) or the production of antioxidant molecules (e.g., ascorbate, glutathione, phenolic compounds, etc. [5]). For instance, TA treatment of *A. thaliana* seedlings was shown to induce the production of scopoletin [51], which is a phytoalexin with antioxidant properties [52]. Additional work will be required to identify how TA treatment leads to a decrease in ROS levels.

These results show that TA-induced PCD can occur in the presence of sub-basal levels of ROS. In animal research, there is increasing evidence that cell proliferation and survival may be compromised not only by toxic ROS levels but also by the lack of necessary ROS, as it could dramatically perturb the cellular redox balance [2,53]. Whether TA can activate PCD by decreasing ROS production down to critically low levels, cannot be excluded at this time.

It was recently shown that TA activates the EDS1/PAD4-mediated defense pathway, which is involved in the induction of plant immunity activated via Toll/Interleukin-1 (TIR)-type immune receptors [38,54]. The EDS1/PAD4-dependent pathway is necessary for the activation of defense responses that may lead to the induction of defense related-cell death, with EDS1 working upstream of the oxidative burst and HR cell death. TA-mediated activation of the EDS1/PAD4 pathway occurred independently of ROS production [38]. Based on these results, the EDS1/PAD4-dependent pathway appears as a possible candidate to mediate TA-induced PCD. In *Arabidopsis* seedlings, TA induces the expression of EDS1/PAD4 dependent genes, such as *FMO1*, *EDS1, PAD4, SAG101*, and *PR1* [38]. Yet, none of these genes were upregulated in *Arabidopsis* cell cultures during the induction of PDC by TA [50], which suggests that the EDS1/PAD4-mediated pathway was not activated during this process. However, we cannot exclude the possibility that these divergent results are due to differences in experimental conditions. Further experiments will be necessary to determine whether the EDS1/PAD4-mediated pathway is involved or not in TA-induced PCD in *Arabidopsis* cell suspensions.

We then investigated whether increasing ROS levels could promote cell survival. To test this hypothesis, we added H_2_O_2_ to cell suspensions to simulate increased ROS production before the addition of TA. The percentage of cell death after TA addition was significantly reduced in cells that had been pre-treated for 3 h with 1 mM H_2_O_2_ (Figure 3)_._ This suggests that reduced H_2_O_2_ production in the presence of TA negatively affects cell survival. This also shows that H_2_O_2_ plays a role in controlling cell sensitivity to TA and its ability to induce cell death. Previous work using the TA resistant mutant *txr1-1* has provided some indirect evidence that increased ROS accumulation may be implicated in the control of resistance to TA in *txr1-1* seedlings [36,55]. The *txr1-1* mutant (also known as *Atpam16-2*) is mutated in the mitochondrial gene *AtPAM16*, which codes for a mitochondrial protein that works as a negative regulator of ROS production [55]. The *Atpam16-1* mutant and TA resistant *txr1-1*/*Atpama16-2* mutant show overproduction of ROS in leaf tissues and enhanced immunity [55]. However, how ROS would modulate TA toxicity has not yet been investigated.

In this work, we found that addition of H_2_O_2_ was efficient in protecting against TA when added 3 h prior to TA treatment. The fact that H_2_O_2_ added to cell suspensions is rapidly degraded, with a half-life of 2 to 5 minutes [56], indicates that the protective effect did not result from the direct interaction of H_2_O_2_ and TA molecules but was due to molecular or biochemical changes induced by H_2_O_2_. We have shown previously that TA-mediated induction of PCD depends on TA’s ability to inhibit cellulose synthesis, since isoxaben, which is another cellulose biosynthesis inhibitor, also activates a similar PCD [27,50]. Hence, TA-induced cell wall damages and alteration in cell wall integrity are involved in the induction of PCD. Apart from its role as a signaling molecule, H_2_O_2_ can activate MAPK signaling cascades, redox-sensitive enzymes, and transcription factors [6]. It can also modify the plant cell wall. In particular, H_2_O_2_ is involved in the peroxidase-mediated cross-linking of cell-wall polymers that leads to cell wall stiffening [12,13]. Production and assembly of secondary cell wall components, such as lignin and suberin, also depend on H_2_O_2_ [57,58]. These changes were proposed to maintain cell wall integrity that can be compromised by cell wall damages, such as those induced by inhibition of cellulose synthesis [59]. Accordingly, we hypothesized that TA and H_2_O_2_ may both cause changes in the cell wall rigidity that may be decisive for cell survival. 

We used AFM–based force microscopy to measure changes in cell wall stiffness induced by TA and H_2_O_2_ in *Arabidopsis* cell suspensions, as described before [46]. In comparison to control cells, TA-treated cells presented an important decrease in cell wall stiffness correlating with inhibition of cellulose biosynthesis (Figure 4a,c). In contrast, the addition of 1 mM H_2_O_2_ increased cell wall stiffness (Figure 4b), with half of Young’s modulus values over 0.5 MPa compared to 29% for control cells and 9% for TA-treated cells. Cell wall stiffening induced by H_2_O_2_ may be the result of peroxidase-mediated cross-linking of various compounds, such as polysaccharide-linked ferulates (bound to lignin or not), extensins, and lignin monomers [13,60]. It was suggested that changes in H_2_O_2_ concentration may shift peroxidase activity from an extensin cross-linking activity to another function [61]. The degree of pectin methylesterification is another key element in the control of cell wall stiffness. H_2_O_2_ can also induce changes in pectin content and de-methylesterification [14], which suggests that H_2_O_2_ mediated pectin modifications may also contribute to cell wall stiffening [62].

The H_2_O_2_-mediated increase in cell wall rigidity was not altered by the subsequent addition of TA (Figure 4d). This indicates that H_2_O_2_ induced changes in the cell wall organization or composition that prevented or compensated for cell wall mechanical alterations induced by TA. Consequently, we suggest that reinforcement of the cell wall mediated by H_2_O_2_ may, at least partially, halt the induction of cell death in response to TA either by protecting cells from cell wall damage or by maintaining cell wall integrity. However, we cannot exclude the possibility that the signaling role of H_2_O_2_ also contributes to the inhibition of TA-induced PCD. Additional studies will be required to evaluate these possibilities. 

In the context of a plant-pathogen interaction, reduction in ROS production by TA may be useful for the pathogen to promote infection. *S. scabies* infects young developing potato tubers mainly through wounds, lenticels, or directly through cells of the nascent periderm [31,35,63]. Secretion of TA perturbs plant cell wall synthesis and reduces ROS production, which decreases overall cell wall rigidity. This, in turn, could facilitate *S. scabies* direct entry through cells. As a defense response against *S. scabies* infection, potato tubers synthesize new cork-like cells embedded with suberin to slow down *S. scabies* invasion. Since ROS are necessary for the assembly of suberin [58,64], inhibiting ROS production may delay the construction of the suberin barrier that protects potato tubers against *S. scabies*, which increases infection.

## 4. Material and Methods

### 4.1. Plant Material and Treatments

Cell suspension cultures of *A. thaliana* accession Landsberg *erecta* derived from stem explants (line PDB-D, *Arabidopsis* Biological Resource Center-ABRC) were graciously provided by Dr. Jean Rivoal [65]. Unless otherwise indicated, all chemicals were purchased from Sigma Aldrich. Cell suspensions were grown in 45 mL Murashige and Skoog (MS) medium (pH 5.7) supplemented with B5 vitamins and 1 mg L^−1^ 2,4-dichlorophenoxyacetic acid (2,4-D) in 125 mL Erlenmeyer flasks kept on a rotary shaker (120 rpm) at 22 °C in the dark. *Arabidopsis* cell cultures were sub-cultured weekly by diluting 15 mL cells into fresh medium. Treatments were performed using 10 mL log-phase cells 3 to 4 days after subculture. Thaxtomin A (TA) was prepared as described before [27]. TA (stock of 1 mM) was prepared in methanol and added at a final concentration of 1 µM. The same volume of methanol (0.1% of final volume) was added to the control cells. H_2_O_2_ was diluted in water at a concentration of 1 M and added to cell suspensions at a final concentration of 1 or 10 mM. To investigate the effect of H_2_O_2_ on the induction of cell death by TA, H_2_O_2_ was added 0, 3, or 6 h prior to TA treatment. 

### 4.2. Cell Death Assay

Cell death was assessed using trypan blue staining, as described before [27]. For each experimental condition, at least 500 cells were counted. Each experiment was repeated at least three times.

### 4.3. ROS Visualization in Cell Cultures

For microscopic evaluation of ROS production, cells were incubated for 30 min in a phosphate buffer solution (PBS) containing 10 µM H_2_DCFDA (2’,7’-dichlorodihydrofluorescein diacetate) and then washed twice with PBS before mounting in medium containing 2.78 µM propidium iodide (PI, [66]). Images were taken with a Zeiss Imager Z1 microscope (Carl Zeiss Canada Ltd., Ontario, Canada) equipped with a monochromatic camera using AxioVision 4.8.2 version. Aliquots of 40 µL of cell culture were examined using an excitation wavelength of 488 nm (H_2_DCFDA) and 535 nm (PI). H_2_DCFDA fluorescence was detected in the presence of ROS in cells with an FITC filter (520 nm). Dead cells labelled by PI were visualized using a rhodamine filter (617 nm). 

### 4.4. Quantification of H_2_O_2_

Cell cultures were treated for 24 h with 0.1% methanol (control), TA (10 µM), or heated at 45 °C for 30 min (positive control [19]). Quantification of H_2_O_2_ in cell extracts was performed using a modified ferrous ammonium sulphate/xylenol orange (FOX) method [43,44]. Samples of filtered cell cultures (0.1 g) were powdered in liquid nitrogen and homogenized in a balanced salt solution [44]. After centrifugation, 100 µL of supernatant were mixed with 0.9 mL of reaction solution (250 mM ammonium ferrous sulphate, 100 mM xylenol orange, and 100 mM sorbitol in 25 mM H_2_SO_4_) and incubated at room temperature for 30 min. Absorbance at 560 nm was determined. Concentration of H_2_O_2_ was calculated using a standard curve and expressed as the equivalent µmol of H_2_O_2_ per mg of cells (fresh weight).

### 4.5. Antioxidant Enzymes Activity Assays

Catalase (CAT) activity was assayed using a protocol modified from Aebi, 1984 [67]. Additionally, 0.1 g of filtered cell cultures were ground in liquid nitrogen and resuspended in 1.5 mL homogenization buffer (50 mM Tris-HCL, 0.1 mM EDTA, 0.2% TritonX-100, 1 mM PMSF, 2 mM DTT). After centrifugation, 70 µL aliquots were taken from the supernatant. Each aliquot was added to a quartz cuvette containing 920 µL assay buffer (50 mM sodium phosphate buffer, pH 7.0, 0.1 mM EDTA). The reaction was initiated by adding 10 µL of 3% (*v*/*v*) H_2_O_2_ or 10 µL of ddH_2_0 (blank). Absorbance at 240 nm was measured every 10 s for 3 min to quantify the decrease in concentration of H_2_O_2_. Enzyme activity was calculated using the H_2_O_2_ extinction coefficient (E) = 39.4 M^−1^ cm^−1^.

Ascorbate peroxidase (APX) activity was determined using a protocol adapted from Nakano and Asada, 1981 [68]. Furthermore, 0.1 g of filtered cell cultures were ground in liquid nitrogen and resuspended in 1.5 mL extraction buffer (50 mM sodium phosphate buffer, pH 7.0, 2% polyvinylpolypyrrolidone (PVPP), 0.1 mM ethylenediaminetetraacetic acid (EDTA), 2 mM ascorbate). After centrifugation, 100 µL aliquots were taken from the supernatant. Each aliquot was added to a quartz cuvette containing 600 µL of 50 mM potassium phosphate buffer, 100 µL 1 mM EDTA, and 100 µL ascorbate. The reaction was started by adding 100 µL of 0.1 mM H_2_O_2_ or 100 µL ddH_2_0 (blank). Absorbance was measured continuously for 3 min at 290 nm to evaluate the reduction in ascorbate. APX activity was calculated using the extinction coefficient of ascorbate (E) = 2.8 mM^−1^ cm^−1^.

To measure superoxide dismutase (SOD) activity, we used a modified protocol from Beauchamp and Fridovich, 1971 [69]. Furthermore, 0.1 g of filtered cell cultures were ground in liquid nitrogen and resuspended in 1.5 mL homogenization buffer (50 mM sodium phosphate buffer, pH 7.8, 1 mM EDTA, 2% (*w*/*v*) PVPP). After centrifugation, aliquots of 200 µL were taken from the supernatant. The reaction mixture (50 mM phosphate buffer, pH 7.8, 33 µM nitro-blue tetrazolium (NBT), 10 mM L-methionine, 0.66 mM EDTA, 0.0033 mM of Riboflavin added just before the assay) was filtered and kept in the dark. Each aliquot was added to a final volume of 3.2 mL of the reaction mixture. Samples were prepared and kept in the dark until all sets were ready. Control and samples were illuminated with a luminescent lamp for 10 min before reading the absorbance at 560 nm. The extinction at 560 nm was read against the blank. One unit of SOD was defined as the amount of sample causing a 50% inhibition of the photoreduction.

### 4.6. Cell Surface Mechanics

The elastic modulus (Young’s modulus) of individual cells was quantified from force/tip-sample separation curves recorded using atomic force microscopy (AFM). Cell cultures were pre-treated for 24 h with 0.1% methanol (control), TA, H_2_O_2_, or a combination of both, as described above. Turgescent cells kept in liquid culture medium were used for this analysis, since plasmolysis can mimic the cellular response to cell wall damage [70,71] and alter the induction of PCD. A 40 µL-aliquot of cells was laid on a poly-l-lysine (0.1 mg mL^−1^) coated slide cover slip for 5 min to allow adhesion. The cover slip was washed three times with culture medium and fixed with a minute drop of vacuum grease in a small petri dish containing 2 mL of culture medium. AFM analysis was conducted in contact mode as described before [46] using a JPK instrument NanoWizard^®^ 4 V.6 (Berlin, Germany) mounted on top of an inverted Zeiss imager Z1 microscope (Carl Zeiss Canada Ltd., Ontario, Canada). We used the MLCT cantilever A with a nominal spring constant of 0.07 N/m (Bruker AFM probes, Camarillo, CA, USA). We obtained spring constants for this cantilever typically ranging from 0.05 to 0.11 N/m using the thermal noise technique [72]. We calculated the Young’s modulus using the Hertz model adapted for a four-sided pyramid indenter, built-in the JPK analysis software. The Young’s modulus of the glass cover slip was considered infinitely rigid when compared to that of the measured cells. All studies were carried at room temperature. Force-curves were recorded at five different arbitrary locations in a 5-μm radius over the cell surface leading to five batches of 30 to 50 points per cell. We used 3 to 4 cells per experiment and each experiment was repeated 3 times.

### 4.7. Statistics

Statistical analysis was performed with GraphPad Prism 7. For the measurements of cell death, at least 500 cells were counted in groups of 100 or 500 cells for one experiment. Each experiment was repeated at least three times. Data was analyzed using the *t*-test or one-way ANOVA for each time point, as indicated. Results were considered statistically different when the *p*-value was <0.05.

## 5. Conclusions

In *Arabidopsis* cell cultures, TA-induced cell death was associated with a decreased ROS accumulation that was not due to the activation of antioxidant CAT, APX, and SOD enzyme activity. However, other proteins or compounds could be involved in scavenging ROS after TA treatment. Perturbation of ROS production by TA may alter cellular redox homeostasis or activate signaling pathways that induce cell death. It can also prevent various H_2_O_2_-dependent cell wall modifications known to strengthen the cell wall. Hence, when exogenous H_2_O_2_ was added to cells prior to TA-treatment, the level of cell death was significantly decreased, which suggests that TA’s inhibitory effect on the production of ROS may contribute to the induction of cell death. While H_2_O_2_ treatment was shown to increase cell wall stiffness, TA treatment had the opposite effect. Moreover, alleviation of TA-induced cell death by H_2_O_2_ pretreatment was associated with cell wall stiffening. We suggest that H_2_O_2_ protection against TA-induced PCD could be, at least partially, due to the H_2_O_2_-mediated increase in rigidity of the cell wall, which could prevent or compensate for cell wall damages induced by TA. 

## Supplementary Materials

The following are available online at https://www.mdpi.com/2223-7747/8/9/332/s1, Supplementary Figure S1: Effect of 0.1% methanol on *Arabidopsis thaliana* cell suspensions viability. Supplementary Figure S2: Induction of cell death in response to thaxtomin A, H_2_O_2_, and a combination of both.
