# Supplementary material for: Reactive Oxygen Species Alleviate Cell Death Induced by Thaxtomin A in Arabidopsis thaliana Cell Cultures
Source: Plants (Basel). 2019 Sep 6;8(9):332. doi: 10.3390/plants8090332 (PMC6784117; doi:10.3390/plants8090332)
Supplement: Supplementary file 1 [file plants-08-00332-s001.zip › Suppl Fig S1.pdf]

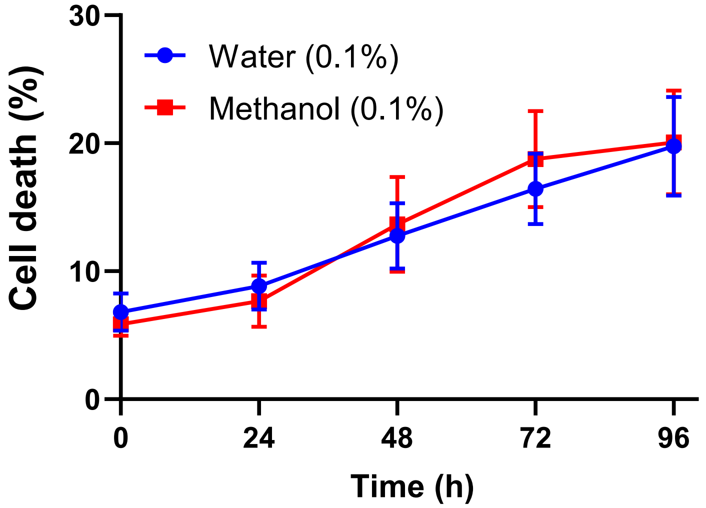

Figure S1. Effect of 0.1% methanol on *Arabidopsis thaliana* cell suspensions viability.

Percentage of cell death in *A. thaliana* cell suspensions treated with water (0.1% final volume) or methanol (0.1% final volume) over a period of 96 hours. Cells were counted in groups of 500. The mean ± SD was calculated from 3 replicates for each time point. Data was analyzed using t-test followed by Holm-Šidák method with alpha = 0.05.
No significant difference ($p < 0.05$) was found for each time point.

Plants **2019**, *8*, 332; doi:10.3390/plants8090332
