# Supplementary material for: Reactive Oxygen Species Alleviate Cell Death Induced by Thaxtomin A in Arabidopsis thaliana Cell Cultures
Source: Plants (Basel). 2019 Sep 6;8(9):332. doi: 10.3390/plants8090332 (PMC6784117; doi:10.3390/plants8090332)
Supplement: Supplementary file 1 [file plants-08-00332-s001.zip › Suppl Fig S2.pdf]

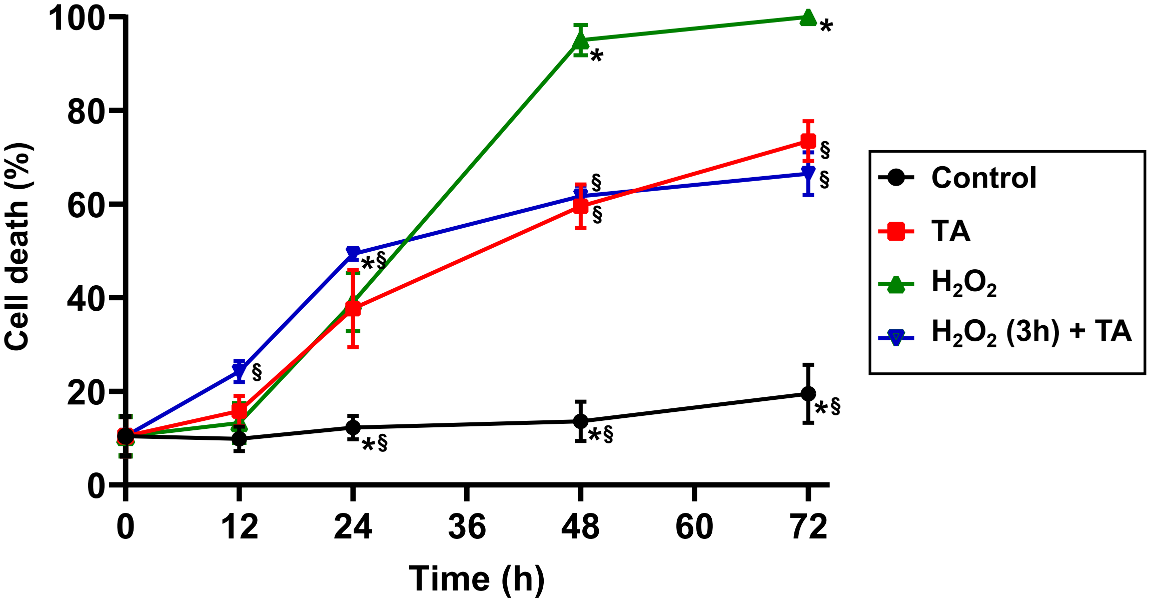

Figure S2. Induction of cell death in response to thaxtomin A, H_2O_2, and a combination of both.

Percentage of cell death in *A. thaliana* cell suspensions at different time points after treatment with 0.1% methanol (Control),1 µM thaxtomin A (TA), 10 mM H_2O_2 (H_2O_2), or with a pretreatment of 10 mM H_20_2 for 3 hours (3h) before adding 1 µM TA. Cells were counted in groups of 100 for a total of 500 cells per experiment. The mean ± SD was calculated from at least 3 experimental replicates for each time point. Data was analyzed using one-way ANOVA. For each time point, a significant difference with TA treatment is indicated by (*) and a significant difference with H_2O_2 treatment is indicated by (§) ($p < 0.05$).

Plants **2019**, *8*, 332; doi:10.3390/plants8090332
